# Comparison of ankle dorsiflexion using XROMM and external angular kinematics in a quadrupedally walking macaque

**DOI:** 10.1242/jeb.251088

**Published:** 2025-09-04

**Authors:** Jeffrey K. Spear, Sharon Kuo, Nicholas J. Gidmark, Michael C. Granatosky, Myra F. Laird, Zewdi J. Tsegai, Carol V. Ward, Callum F. Ross

**Affiliations:** ^1^ Department of Organismal Biology and Anatomy, University of Chicago, Chicago, IL 60637, USA; ^2^ Department of Biomedical Sciences, University of Minnesota, Duluth, MN 55812, USA; ^3^ Technological Primates Research Group, Max Planck Institute for Evolutionary Anthropology, 04103 Leipzig, Germany; ^4^ Department of Biology, Knox College, Galesburg, IL 61401-4999, USA; ^5^ Department of Anatomy, New York Institute of Technology College of Osteopathic Medicine, Old Westbury, NY 11568, USA; ^6^ Center for Biomedical Innovation, New York Institute of Technology College of Osteopathic Medicine, Old Westbury, NY 11568, USA; ^7^ Department of Basic and Translational Sciences, University of Pennsylvania, Philadelphia, PA 19104-6030, USA; ^8^ Department of Pathology and Anatomical Sciences, University of Missouri, Columbia, MO 65201, USA

**Keywords:** Biomechanics, Foot, Locomotion, Primates

## Abstract

Movement at complex joints, such as the ankle, can be challenging to quantify from external kinematics alone. We compared X-ray reconstruction of moving morphology (XROMM) and angles derived from high-speed video footage to study dorsiflexion in the ankle of a rhesus macaque (*Macaca mulatta*) during stance phase. We found inconsistent correspondence between angles measured on standard videos using five approaches and those measured directly on the talocrural joint using XROMM, indicating that different measurement methods capture different kinematic processes. Many externally measured angles indicate a range of motion of 30 deg during stance phase. The XROMM data, however, demonstrate that the talocrural joint itself only dorsiflexes about 15 deg through stance phase, with much greater mobility during swing phase. This suggests that other parts of the foot, likely the midfoot, contribute substantially to dorsiflexion in stance phase, while the talocrural joint is actively held in place.

## Teaser

**Summary:** Different approaches to measuring ankle dorsiflexion capture different underlying kinematics. Evidence indicates that dorsiflexion at the talocrural joint is actively limited during walking in a macaque.

## INTRODUCTION

Understanding an animal's movement is critical to identifying the selective forces that shaped its evolution (Granatosky, 2018; Hunt et al., 1996; Preuschoft, 2002). Primates are well adapted for navigating complex arboreal environments, and locomotor biomechanics, both kinetic and kinematic, have been explored across a range of taxa and substrate types (e.g. D'Août et al., 2002; Greiner and Ball, 2014; Janisch et al., 2024; Reghem et al., 2013; Wright et al., 2008). However, these efforts often rely on video footage of primates navigating natural or naturalistic environments and measuring joint angles at specific points in the gait cycle. These kinematic measurements are then used as proxies for individual joint movements. Direct measurements of *in vivo* kinematics at specific joints using fluoroscopic videos in primates are limited (e.g. Jenkins, 1972; Jenkins and Fleagle, 1975; Jenkins et al., 1978; Kuo, 2020). This hinders our ability to understand comparative biomechanics at specific joints and to draw robust conclusions about the biomechanical importance of fossil morphologies, especially when those fossils represent only part of a larger joint complex.

How well external measurements capture internal movements and relationships among bones during activity remains unclear, especially for complex joint systems like the ankle (Berillon et al., 2010; DeSilva, 2008, 2009; Finestone et al., 2018; Holowka et al., 2017; Meldrum, 1991; Zeininger et al., 2020). The ankle is composed of two distinct joints: the talocrural joint (between the tibia and the talus) and the subtalar joint (between the talus and the calcaneus). In primate research, externally measured ankle angles sometimes also incorporate information from the midfoot and forefoot (DeSilva, 2009; Zeininger et al., 2020), further complicating inferences about movement at specific joints. While in humans there is good correspondence between angles at the ankle measured with marker-assisted optical motion capture and those measured more directly using video radiography (Kessler et al., 2019), how well this translates to kinematic measurements in the less formalized settings typical of most non-human primate studies is unknown. Most primate species have more mobile foot and ankle joints than do humans, however (e.g. Berillon et al., 2010; DeSilva, 2010; Venkataraman et al., 2013). In Afro-Eurasian monkeys in particular, the midfoot, rather than the ankle itself, is thought to contribute substantially to dorsiflexion throughout stance phase (DeSilva, 2008; Meldrum, 1991).

In this study, we compared dorsiflexion across the stance phase of multiple steps from a single rhesus macaque, *Macaca mulatta*, while it walked on a treadmill. We examined dorsiflexion of the foot and ankle captured with high-speed cameras using five approaches to measuring ankle kinematics from light videos. Because these methods are frequently interpreted as reflecting mobility at the talocrural joint specifically (Berillon et al., 2010; DeSilva, 2009; Venkataraman et al., 2013), we also compared these methods with X-ray reconstruction of moving morphology (XROMM) at the talocrural joint. We asked two questions: (1) are markerless kinematics based on video footage a useful proxy for talocrural joint kinematics at the ankle in a non-human primate?; and (2) how does the talocrural joint contribute to foot kinematics throughout stance phase?

## MATERIALS AND METHODS

### Specimen

One adult, captive-raised male macaque, *Macaca mulatta* (Zimmerman 1780), was used in this study. This research specimen was previously included in a feeding study at the University of Chicago. All protocols were approved by the University of Chicago Animal Care and Use Committee and complied with the National Institutes of Health Guide for the Care and Use of Laboratory Animals (ACUP 71565). During the surgical procedure to place markers, the animal was sedated using an intramuscular injection of ketamine and dexmedetomidine and continuously monitored by the University of Chicago veterinary team. Non-bioreactive tantalum beads were surgically implanted into the cortical bone using a 1 mm hand drill. Four markers were implanted in the right tibia, and four in the right talus. After surgery, the macaque could walk immediately but was given over 2 weeks to recover prior to experimentation. Tantalum beads are radio-opaque and create identifiable markers that can be registered between computed tomography (CT) scans and biplanar videofluoroscopy images (Brainerd et al., 2010). This approach is standard in XROMM studies (e.g. Brainerd et al., 2016; Gidmark et al., 2014; Laird et al., 2023; Li et al., 2023; Menegaz et al., 2015; Orsbon et al., 2020; Tsai et al., 2020) and has also been performed in humans (Lundberg, 1989) with reported discomfort lasting a maximum of 1 week.

### Experimental procedure

The macaque was trained to walk on a treadmill inside a polycarbonate box using positive reinforcement (e.g. Druelle and Molina-Vila, 2021; Schapiro et al., 2003). The treadmill was set to run at 1.1 miles per hour (0.49 m s^−1^). Data were collected across 2 days in the XROMM facility at the University of Chicago. Two cameras recorded radiographic videos created by sending X-rays from a pair of fluoroscopes at oblique angles through the animal's legs to image intensifiers (90–110 kV, 10–12.5 mA); one camera recorded standard high-speed images in the visible light spectrum. Videos were recorded at 150 frames s^−1^. Two different setups were used for the visible light camera, but only videos taken perpendicular to the treadmill were used in the analysis.

CT scans of the lower limb were collected at the University of Chicago using a Vimago Robotic HDC scanner from Epica Medical Innovations (80 kV, 60 mA for 7 ms at a resolution of 300 μm). The scans were segmented in 3D Slicer version 5.6.2 (Fedorov et al., 2012) to create three-dimensional polygonal models of both bones and the tantalum markers.

### Data processing

Clarity and framing of the foot in the light and fluoroscopy images through the stance phase of each step were assessed to determine suitability for inclusion. For the light images, the heel, knee and metatarsophalangeal joint had to be in-frame and visible. For the fluoroscopy images, at least three tantalum beads in both the talus and tibia had to be in-frame and identifiable throughout. Six steps in the light videos and ten steps in the fluoroscope videos were deemed suitable for inclusion; all visible light videos are from the first day, and all fluoroscope videos are from the second. All but one step used a diagonal sequence gait (Table S1).

**Fig. 1.**
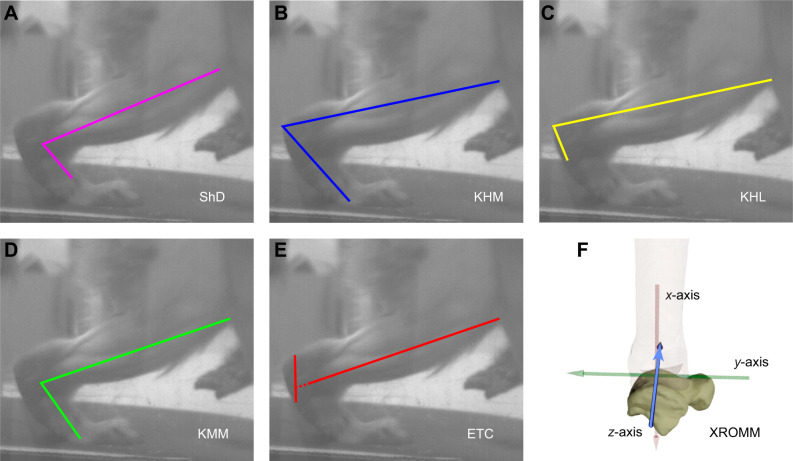
**Six methods of measuring ankle dorsiflexion.** (A) Shank to dorsum of the foot (ShD); (B) knee to heel to fifth metatarsal head (KHM); (C) knee to heel to lateral margin of the foot proximal to midfoot bend (KHL); (D) knee to medial malleolus to fifth metatarsal head (KMM); (E) angle between a line between the knee and the medial malleolus and a line between the superior posterior calcaneus and the intersection of the midfoot and hindfoot planes (ETC); and (F) X-ray reconstruction of moving morphology (XROMM) joint coordinate system. The *z*-axis (blue) is dorsiflexion.

Dorsiflexion in visible light images was estimated using five different methods (Fig. 1A–E): (1) as the angle between the shank and the dorsum of the foot (shank–dorsum, or ‘ShD’ method), following Zeininger and colleagues (2020); (2) as the angle from the knee to the heel to the head of the fifth metatarsal (knee–heel–metatarsal, or ‘KHM’ method), following DeSilva (2008, 2009) and Venkataraman and colleagues (2013); (3) as a modification of method 2, where the second line follows the skin/hair line on the lateral side of the foot (per DeSilva, 2009) but does not end at a particular landmark (knee–heel–lateral, or ‘KHL’ method); (4) as the angle from the knee to the medial malleolus and from the medial malleolus to the head of the fifth metatarsal (knee–malleolus–metatarsal, or ‘KMM’ method), following Berillon et al. (2010; their ‘ankle angle’); and (5) as the angle between a line drawn between the knee and the medial malleolus and a second drawn from the posterior–superior margin of the calcaneus to a point at the intersection of the axis of the hindfoot and midfoot (external talocrural or ‘ETC’ method), following Berillon et al. (2010; their ‘talocrural angle’). Angles were measured in Fiji version 1.54k (Schindelin et al., 2012). Each frame in the step was measured twice by a single author (J.K.S.) to assess and minimize intra-observer error, and the average angle for each frame was used. To reduce noise from measurement error, data were passed in both directions through a second-order low-pass Butterworth filter (resulting in a zero-lag, fourth-order filter) using the butter and filtfilt functions in the signal package version 1.8-1 (https://r-forge.r-project.org/projects/signal/) in R version 4.3.3 (http://www.R-project.org/). Cutoff frequency was set individually for each time series using residual analysis (Winter, 2009; see Table S2).

Dorsiflexion in X-ray fluoroscopy images was assessed using XROMM (Brainerd et al., 2010). The locations of tantalum markers were tracked in XMALab version 2.1.0 (Knörlein et al., 2016). The constellation of markers relative to the rest of the bone was calculated in Maya 2025 (https://www.autodesk.com/) using vertex averaging from the MEL-coded plugin for XROMM animation (Brainerd et al., 2010). The relative three-dimensional position of markers was imported into XMALab to calculate rigid body transformations. Three-dimensional models of each bone were then imported into Maya and animated using the rigid body transformations of each bone. A joint coordinate system based on anatomical landmarks was assigned to the talocrural joint to quantify rotation at the ankle (Fig. 1F; Fig. S1). All XROMM trials were combined into a single animation event with the same joint coordinate system.

### Data analysis

Angle data were exported from Maya or Fiji and analyzed in R version 4.3.3 (http://www.R-project.org/). Angles were subtracted from 90 deg so that positive values represent dorsiflexion and negative values represent plantarflexion. Comparisons were conducted using both raw and standardized angles to facilitate comparisons across the entire stance phase using angles measured in different ways. Angles were standardized by translating them such that the average angle at contact for each set of measurements was zero. This moves the data to the same starting point at the beginning of stance phase, while preserving both relative changes and variation across trials at every part of the step.

To compare ankle dorsiflexion in a macaque using different methods, we tested for statistical differences in both absolute and standardized angles, as well as intraobserver error across the five methods of externally measured angles. The maximum dorsiflexion and maximum plantarflexion angles during stance phase were compared across sets of trials using a Kruskal–Wallis test with *post hoc* Wilcoxon rank sum tests. For unstandardized angles, the angle at the start, end and middle of stance phase was also compared.

We measured angular velocities through stance phase by subtracting the angle in each frame of stance phase from the angle in the previous frame and multiplying by the frame rate (150 frames s^−1^) to calculate degrees per second. Dorsiflexion was scored as positive angular velocity and plantarflexion was scored as negative angular velocity.

We compared angles and angular velocities across different methods using statistical parametric mapping (Pataky, 2010), implemented in the spm1d package version M.0.4.10 (Pataky, 2012) in Matlab version 24.1 (MathWorks, Inc., Natick, MA, USA). We used ANOVA with pairwise *post hoc t*-test to compare different methods across stance phase. We followed Rubin (2021) in performing alpha adjustment only for disjunction testing, where a significant difference observed in any one of several tests is sufficient to reject the null hypothesis. Because we would consider a difference in angle or angular velocity measured between different methods as sufficient reason to reject the joint null hypothesis that the different methods produce indistinguishable results, we set alpha in the statistical parametric mapping analyses to 0.025 (0.05/2 tests of a single joint null hypothesis).

## RESULTS AND DISCUSSION

### Comparing methods: repeatability of angle measurements

We first tested the repeatability of the quantification of joint angles based on external kinematics. This intraobserver error analysis demonstrated a clear difference between the KHM method, where the angle is more repeatable as it is defined by three easily visible anatomical landmarks, and the methods without clear landmarks (ShD, KHL and ETC). Both the mean and maximum error rates across trials were significantly higher for the latter methods (ShD: 3.1 deg, 12.7 deg; KHL: 2.7 deg, 14.0 deg; ETC: 3.6 deg, 16.9 deg) than the former (KHM: 1.1 deg and 5.4 deg; *P*=0.002; Table S3). KMM method values fell in the middle (2.0 deg and 10.0 deg). Overall, error rates were broadly comparable to those reported in other studies using similar methods (DeSilva, 2009; Finestone et al., 2018).

### Comparing methods: absolute angles of dorsiflexion and plantarflexion

**Fig. 2.**
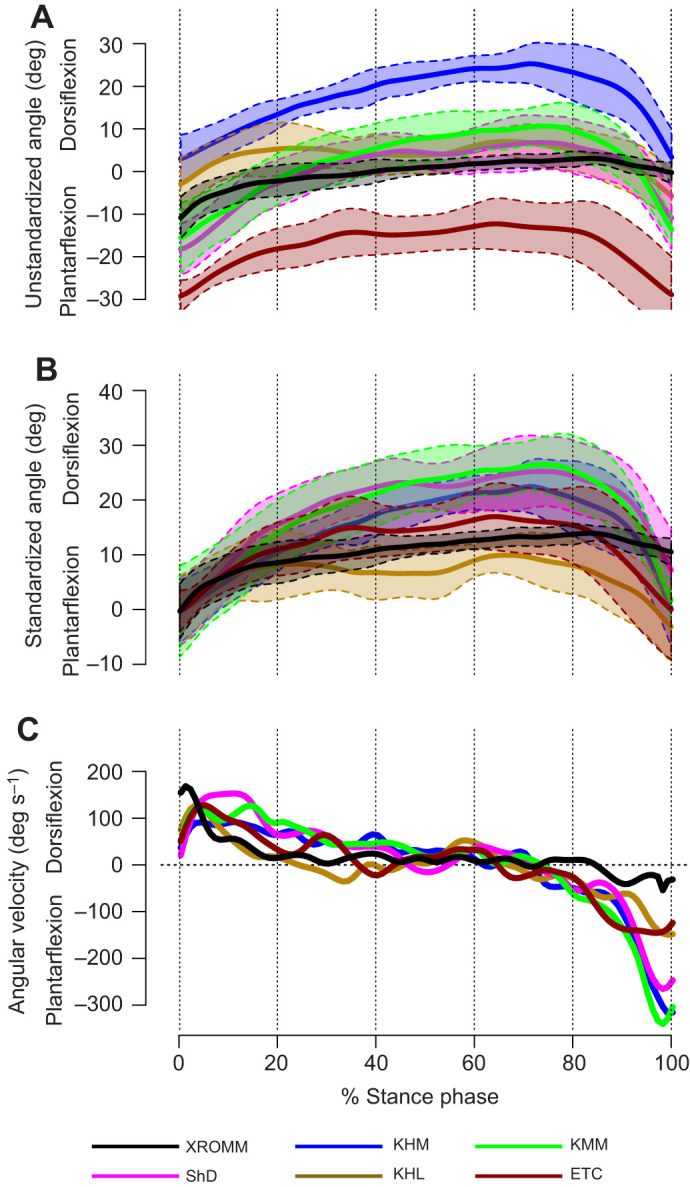
**Results of this study.** (A) Unstandardized angles showing mean and standard deviation of each set of trials. (B) Standardized angles showing mean and standard deviation of each set of trials. (C) Average angular velocity recorded by each method. Negative angular velocities indicate a change toward greater plantarflexion.

Example steps are shown in Movies 1 and 2. The absolute angles quantified during stance phase using the six approaches produced significantly different results (Fig. 2A; Fig. S2; *P*<0.001), with most pairwise comparisons indicating at least some significant differences (Fig. S3). There were differences among methods when measuring maximum dorsiflexion, maximum plantarflexion and dorsiflexion at the beginning, middle and end of stance phase. Most of the pairwise comparisons were significantly different; only the comparison between ShD and KMM values exhibited no significant differences (Table S4).

Statistical parametric mapping also revealed differences among the methods. Values from ShD, KHL and KMM were generally not significantly different from one another in pairwise comparisons except for a small difference between KHL and ShD in the first 10% of stance phase (Fig. S3). KHM measured significantly greater dorsiflexion than all other methods across most of stance phase. ETC measured significantly less dorsiflexion than all other measurements throughout nearly all of stance phase. XROMM measured significantly greater dorsiflexion than ETC and significantly lower dorsiflexion than KHM throughout nearly all of stance phase. XROMM also measured significantly greater dorsiflexion than KMM and ShD at the very end of stance phase, as well as slightly lower dorsiflexion than KHL and KMM through part of the second half of stance phase. That different approaches to measuring ankle dorsiflexion produce significantly different absolute values for ankle dorsiflexion complicates direct comparisons across studies.

### Comparing methods: relative joint movements

It is perhaps unsurprising that different methods of measuring ankle dorsiflexion produce different angles in absolute terms. The KHM method, for example, uses landmarks at the anterior part of the leg proximally and the posterior part of the leg distally, which will naturally form a more acute angle than other methods. Thus, relative joint movements were also compared (Fig. 2B) to assess whether different methods capture similar underlying processes even if they do not measure similar angles. With standardized angles, maximum plantarflexion did not differ significantly across methods, but all external methods except KHL exhibited significantly higher maximum dorsiflexion than did XROMM (Table S4). There were some significant differences in maximum dorsiflexion across the external methods, with KHL and ETC generally having lower maximum dorsiflexion.

Statistical parametric mapping of joint angles showed significant differences between XROMM and all other methods except ETC (Figs S4, S5). For ShD, KHM and KMM, this difference corresponds to the middle of stance phase, in which the three methods measured significantly greater dorsiflexion. For KHL, the difference with XROMM was small and limited to only the end of stance phase (starting at 84%). Indeed, the methods of measuring angles from video footage showed a sharp decrease in dorsiflexion at the end of stance phase, but the drop seen in the XROMM data was more modest. This corresponded with a lower angular velocity (i.e. greater plantarflexion velocity) at the end of stance phase in four of five external methods (excluding KHL) compared with XROMM (Fig. 2C; Figs S6, S7). XROMM also indicated a significantly higher dorsiflexion velocity near the beginning of stance (around 10–15%) compared with ShD or KMM. Overall, KHL and ETC, which attempt to limit angular measurements to only the hindfoot, best approximated the movement of the talocrural joint in a macaque foot, although ETC systematically underestimated dorsiflexion and both methods were prone to error in our trials without visible markers.

### Contribution of the talocrural joint to foot kinematics in a macaque

We found no signal in our XROMM data of joint rotation in either external rotation or inversion (*x*- and *y*-axis; Fig. S8). Dorsiflexion at the talocural joint as measured by XROMM during stance phase was modest, only around 15 deg (Table 1). Angles measured on video footage using the ShD, KHM or KMM methods suggest a range of motion twice that large (Table 1; Table S5). Because these methods capture dorsiflexion and plantarflexion across the whole foot, this result suggests that around half of dorsiflexion observed in the foot of macaques comes from dorsiflexion at the midfoot, whether at the midtarsal joint, the tarso-metatarsal joint, or both (DeSilva, 2010; Elftman and Manter, 1935; Kuo, 2020; Meldrum, 1991; Susman, 1984). This cannot solely be due to the talocrural joint dorsiflexing to its maximum degree before the midfoot takes over (contra Meldrum, 1991), as it dorsiflexes substantially more while unloaded during the swing phase (Table 1). Instead, mobility at the talocrural joint appears to be actively limited through much of stance phase (angular velocity near zero; Fig. 2C; Fig. S9), while the midfoot is allowed to bend. This could represent evidence for elastic energy storage in a mobile midfoot improving energetic efficiency (Bennett et al., 1989; Vereecke and Aerts, 2008). An elastic rebound at the midfoot might also explain the apparent over-correction toward plantarflexion seen toward the end of the stance phase in the externally measured angles compared with the XROMM data (Fig. 2), as well as the sharp increase in angular velocity of plantarflexion in the three whole-foot measurements (ShD, KHM and KMM; Fig. 2C; Fig. S9).

**Table 1.** Summary of dorsiflexion, plantarflexion and range of motion from each method

		XROMM	ShD	KHM	KHL	KMM	ETC
Maximum dorsiflexion (stance)	Minimum	1.5	5.3	22.0	4.9	7.5	−22.4
	Maximum	7.8	16.6	33.7	14.8	19.4	−6.9
	Mean	4.0	9.9	26.6	9.7	13.2	−10.9
Maximum plantarflexion (stance)	Minimum	−2.0	−10.7	6.6	−2.3	−12.6	−28.0
	Maximum	−19.7	−24.9	−9.5	−16.2	−24.6	−45.7
	Mean	−10.8	−20.5	−0.5	−7.8	−19.1	−33.0
ROM (stance)	Minimum	9.7	20.4	18.9	13.0	26.6	18.1
	Maximum	21.2	35.7	31.9	21.9	39.1	26.1
	Mean	14.8	30.4	27.2	17.5	32.3	22.2
Maximum dorsiflexion (all measured)	Minimum	3.5	8.1	22.0	4.9	11.1	−22.4
	Maximum	23.3	18.0	33.7	23.4	19.4	−3.8
	Mean	11.8	14.1	27.5	16.0	13.8	−9.8
Plantarflexion (all maximum measured)	Minimum	−7.9	−13.6	0.2	−5.7	−18.5	−28.0
	Maximum	−20.5	−29.7	−17.6	−19.6	−25.1	−46.0
	Mean	−14.4	−21.9	−6.0	−10.6	−21.9	−34.1
ROM (all measured)	Minimum	17.0	31.5	29.3	20.6	31.8	18.3
	Maximum	43.9	40.7	39.6	36.2	42.0	30.3
	Mean	26.2	36.0	33.5	26.6	35.8	24.3
Start of stance	Minimum	−19.7	−24.0	−3.5	−7.1	−24.6	−33.1
	Maximum	−2.0	−10.7	13.0	9.8	−2.5	−22.5
	Mean	−10.8	−18.2	3.0	−2.9	−15.5	−29.2
Midstance	Minimum	−1.6	−0.1	18.5	−0.2	1.0	−22.6
	Maximum	4.7	9.5	28.1	12.2	13.5	−11.4
	Mean	1.1	4.4	22.5	3.8	7.9	−14.5
End of stance	Minimum	−3.5	−24.9	−9.5	−16.2	−20.1	−45.7
	Maximum	3.2	−3.1	9.1	0.0	−11.4	−19.5
	Mean	−0.2	−10.9	3.4	−5.7	−13.6	−28.9

All values in degrees. Negative values indicate plantarflexion. ShD, shank to dorsum of the foot; KHM, knee to heel to fifth metatarsal head; KHL, knee to heel to lateral margin of the foot proximal to midfoot bend; KMM, knee to medial malleolus to fifth metatarsal head; ETC, angle between a line between the knee and the medial malleolus and a line between the superior posterior calcaneus and the intersection of the midfoot and hindfoot planes; XROMM, X-ray reconstruction of moving morphology; ROM, range of motion.

The talocrural joint itself appears predominantly to position the foot prior to contact, with a high degree of dorsiflexion during swing phase transitioning to slight plantarflexion during initial contact with the substrate. The XROMM data indicated a change in dorsiflexion of more than 30 deg immediately prior to touchdown – twice what we observed across the whole of stance phase. This pattern was apparent in all methods of measuring dorsiflexion and plantarflexion (Table 1; Fig. S10), although interestingly only prior to contact; there was much more variability after push-off. The correspondence among methods and trials during late swing phase is striking because this phase was not standardized; however, standardization may not be necessary as swing phase length tends not to be especially variable within a single individual (Arshavskii et al., 1965; Grillner et al., 1979; Halbertsma, 1983; Mendes et al., 2015).

### What is dorsiflexion?

We measured dorsiflexion using six established methodologies, each of which could represent a different underlying biological phenomenon. When discussing ankle dorsiflexion, we typically envisage rotation about a mediolateral axis at the center of rotation between the medial and lateral malleoli, without translation. It is this definition that is precisely captured using the XROMM approach we employed (Fig. 1D; Fig. S1). This definition of dorsiflexion also seems to be captured, to a large extent, by ETC (Berillon et al., 2010), albeit with low precision on an unmarkered animal. Our results make clear, however, that this definition represents only a partial picture of how the foot and ankle move. Externally measured angles capture different relationships among bones and joints, and represent a broader definition of dorsiflexion, i.e. raising and lowering the foot during the gait cycle. ShD, KHM and KMM all seem to capture this definition, which likely includes substantial contributions from the midtarsal and/or tarso-metatarsal joint. Each approach should be recognized as measuring distinct phenomena that might be more biologically meaningful in different contexts. For example, when trying to interpret the consequences of dorsiflexion for talar and distal tibia morphology in a comparative context (e.g. Tsegai et al., 2017), the narrower definition is likely to be most useful. When trying to understand the ability of the foot to rotate in response to varying substrate orientations (e.g. DeSilva, 2008), the broader definition may have more utility. Which approach should be chosen depends on the research questions asked and the practicality of each method, while interpretations must carefully consider what biological phenomena the underlying data represent.

### Limitations

This study included a limited number of steps from a single individual, and optical measurements were taken from different steps to fluoroscopic measurements. Further, although the individual was walking on a treadmill moving at a constant speed, it would frequently accelerate or decelerate, so steps were not all taken at the same speed. Additional research on a larger sample of both individuals and taxa will be valuable to determine whether the patterns observed here are generalizable, and whether there are systematic corrections that can be done to infer ankle dorsiflexion from external videos in macaques. It is also possible that approaches to measuring kinematics with video cameras in three dimensions and/or those leveraging new machine learning technologies could better approximate the three-dimensional measurement used in this study (XROMM), although such an approach may still need to take place in a laboratory setting.

### Conclusions

This study examined the contribution of the talocrural joint to ankle dorsiflexion in a macaque and compared several methods of measuring ankle dorsiflexion from visible light videos with those measured using XROMM. We found that the talocrural joint contributes only partially to dorsiflexion throughout stance phase. Instead, the midfoot appears to be a major driver of dorsiflexion, possibly facilitating elastic energy storage. We also found that many measures of ankle dorsiflexion from visible light images are not comparable to measures made of the talocrural joint using XROMM, indicating that caution is required when studying the angular kinematics of complex joints outside of a formal laboratory setting.

## Supplementary Material

10.1242/jexbio.251088_sup1 Supplementary information
