# Supplementary material for: Comparison of ankle dorsiflexion using XROMM and external angular kinematics in a quadrupedally walking macaque
Source: J Exp Biol. 2025 Sep 4;228(17):jeb251088. doi: 10.1242/jeb.251088 (PMC12450464; doi:10.1242/jeb.251088)
Supplement: Supplementary information [file jexbio-228-251088-s1.pdf]

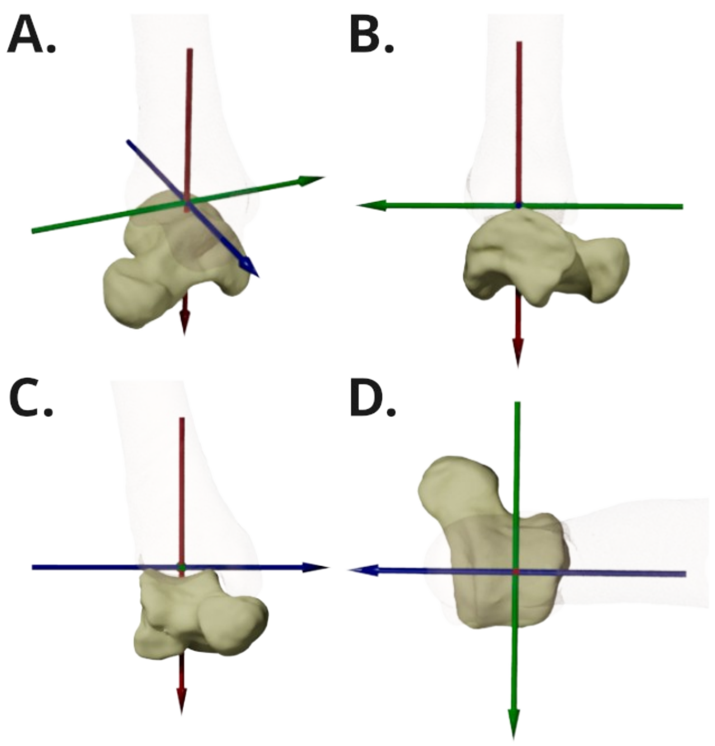

Fig. S1. Joint axis coordinate system for XROMM. The x-axis (red; external rotation) extends up the tibial shaft (albeit at an angle, since the talocrural joint is not perpendicular). The y-axis (green; inversion) follows the trochlear groove. The z-axis (blue; dorsiflexion) is defined by landmarks at the most proximal points of the talar trochlea. A: Oblique view; B: Medial view; C: Distal view; D: Superior view.

Journal of Experimental Biology • Supplementary information

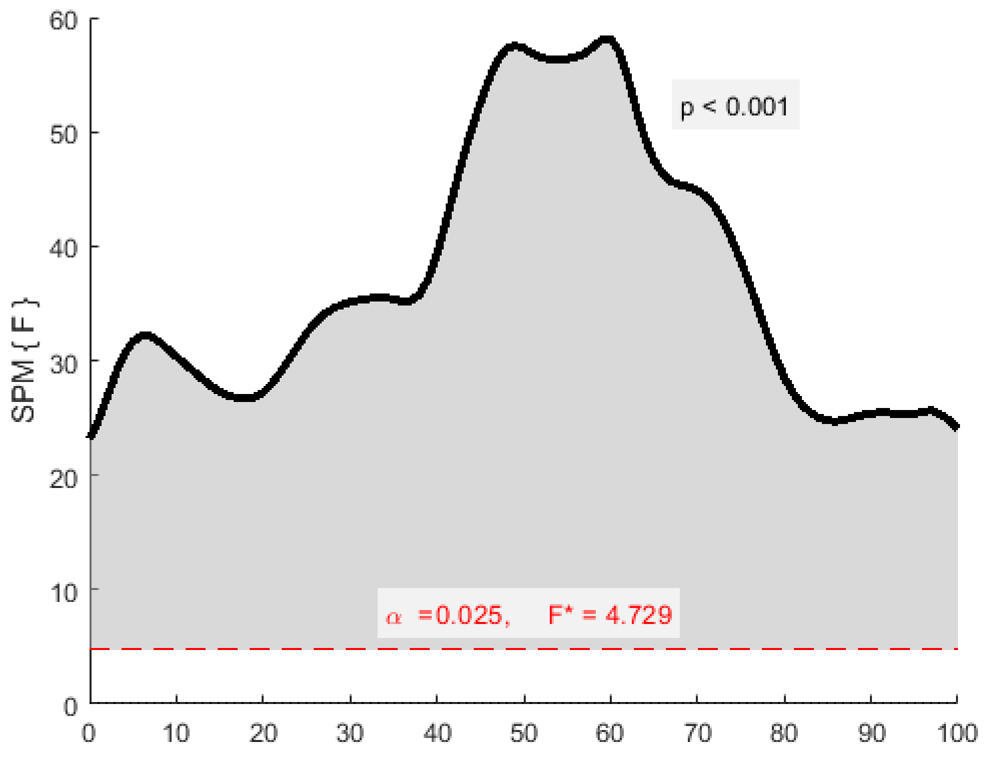

Fig. S2. Statistical parametric mapping ANOVA of unstandardized angles. F statistics above the critical threshold indicate a significant difference among groups at that point of stance phase.

Journal of Experimental Biology • Supplementary information

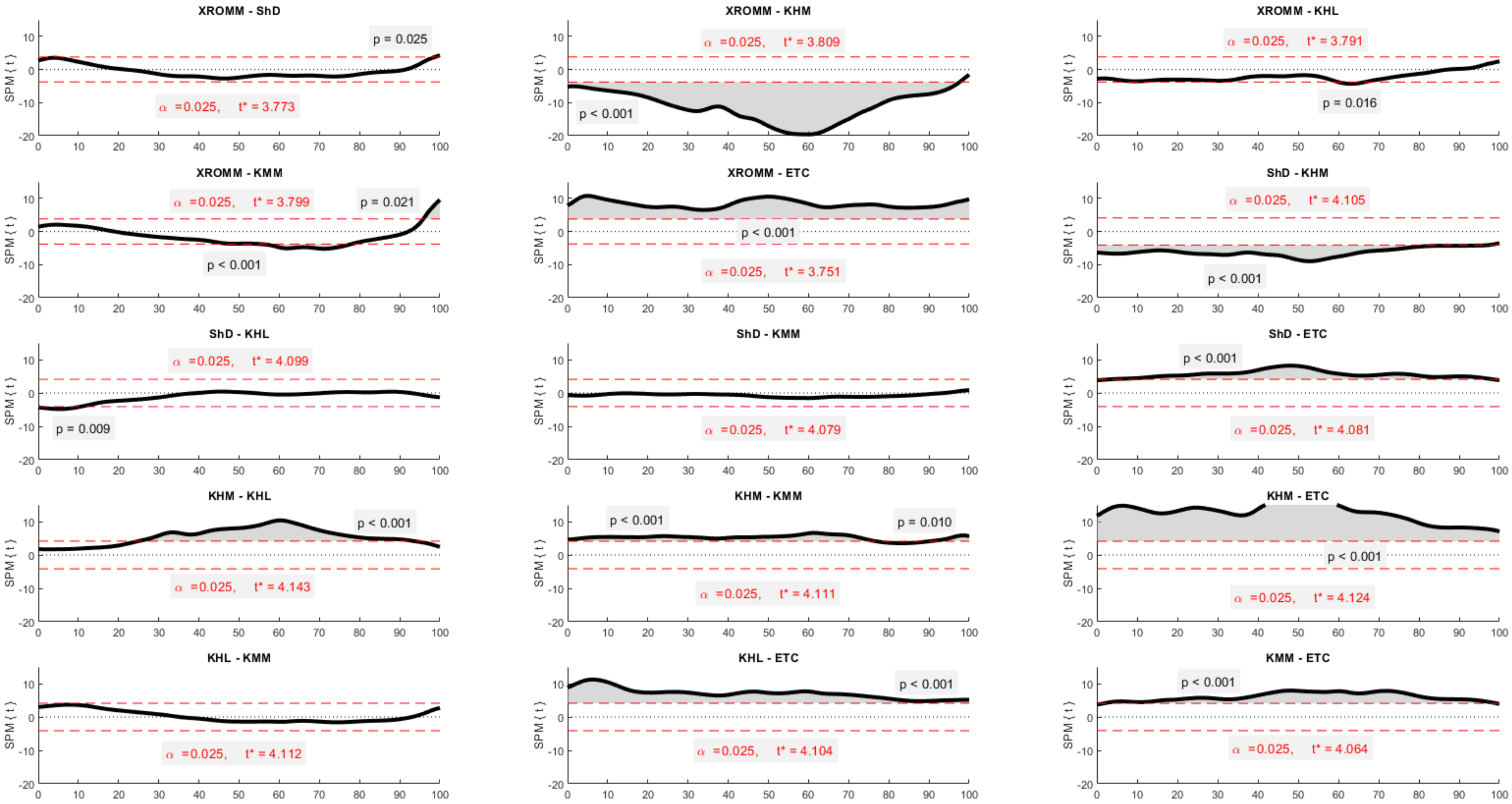

Fig. S3. Pairwise comparisons of each method using statistical parametric mapping with unstandardized angles. Red dashed lines represent critical thresholds for the t statistic for a given alpha level (0.025). Grey areas indicate regions that exceed the critical threshold. T statistics above zero indicate that the first method listed produces larger angles on average.

Journal of Experimental Biology • Supplementary information

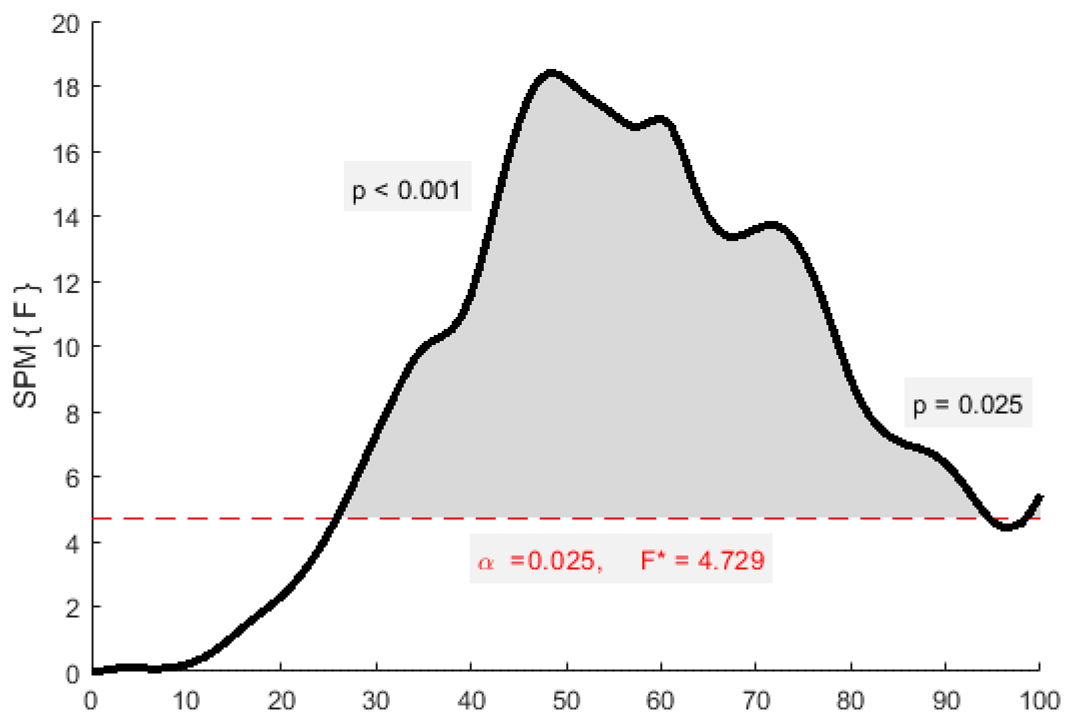

Fig. S4. Statistical parametric mapping ANOVA of standardized angles. F statistics above the critical threshold indicate a significant difference among groups at that point of stance phase.

Journal of Experimental Biology • Supplementary information

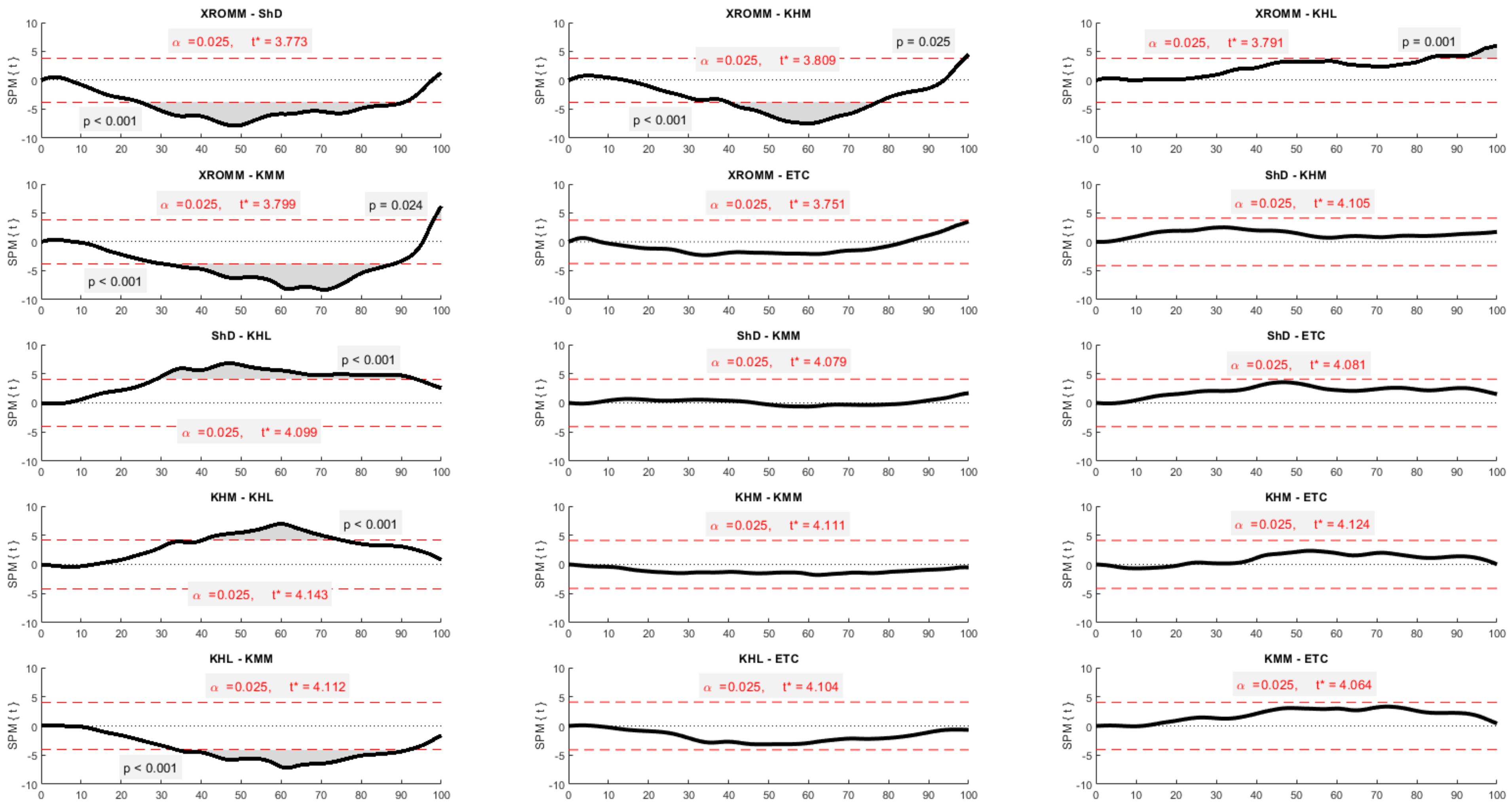

Fig. S5. Pairwise comparisons of each method using statistical parametric mapping with standardized angles. Red dashed lines represent critical thresholds for the t statistic for a given alpha level (0.025). Grey areas indicate regions that exceed the critical threshold. T statistics above zero indicate that the first method listed produces larger angles on average.

Journal of Experimental Biology • Supplementary information

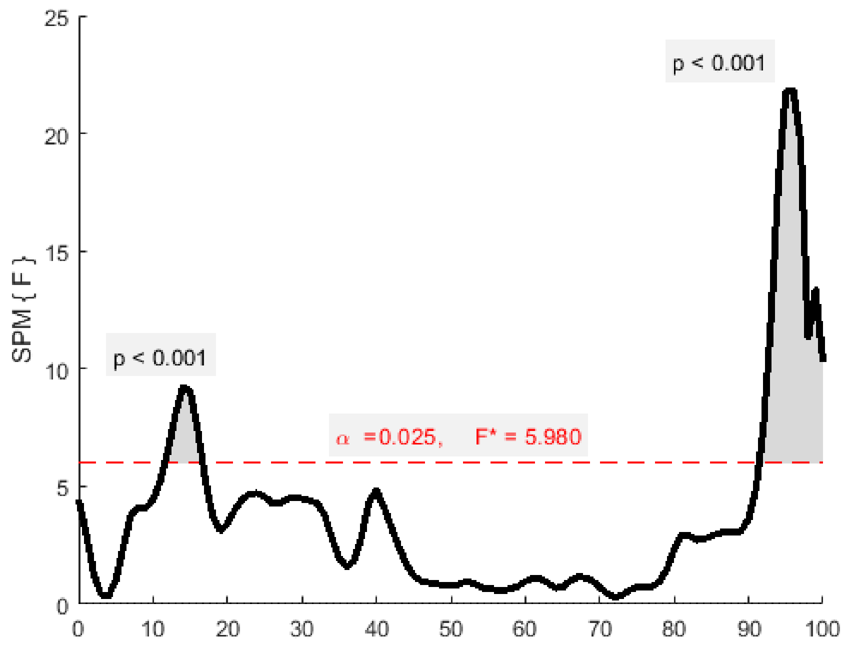

Fig. S6. Statistical parametric mapping ANOVA of angular velocities. F statistics above the critical threshold indicate a significant difference among groups at that point of stance phase.

Journal of Experimental Biology • Supplementary information

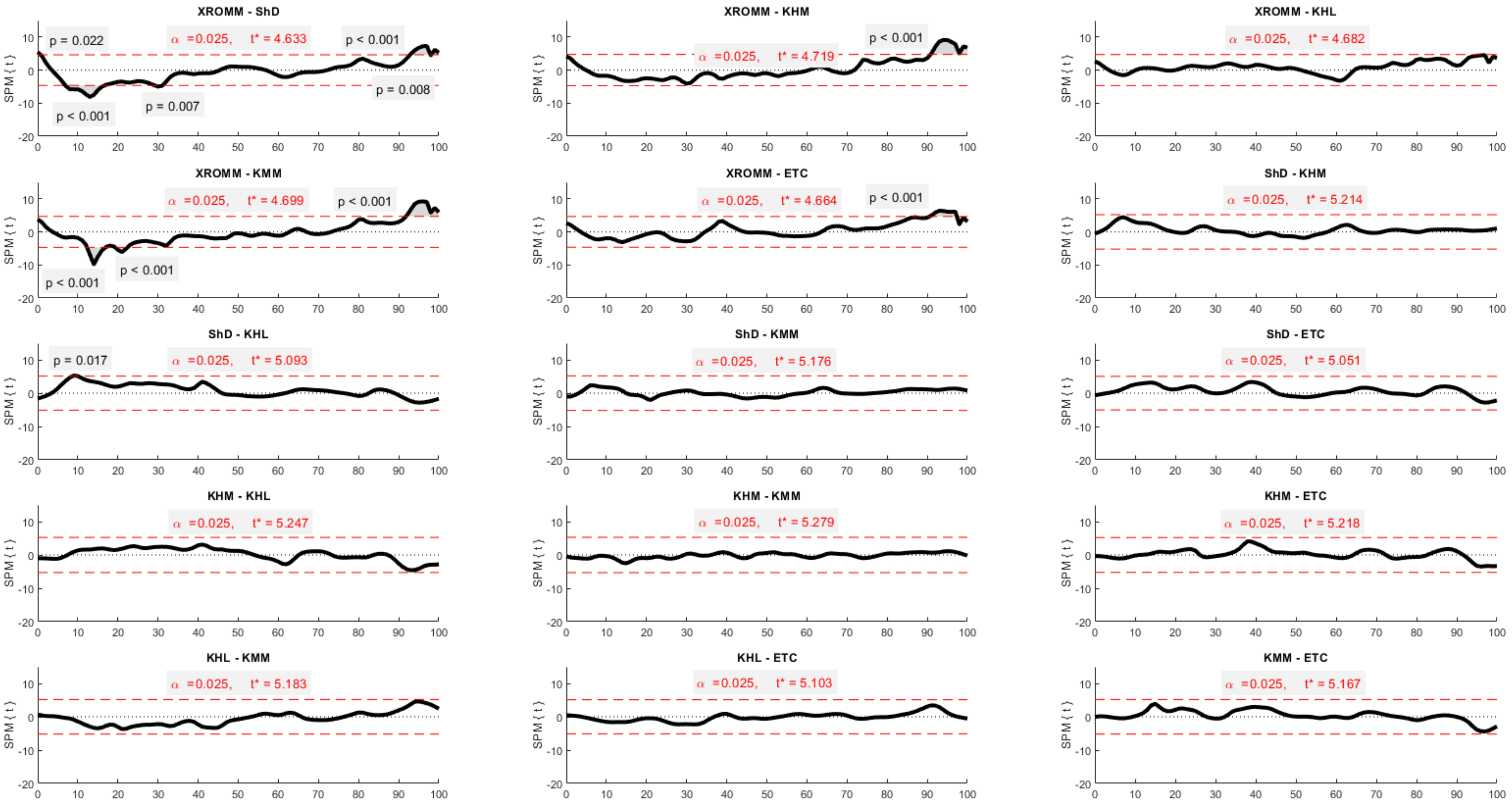

Fig. S7. Pairwise comparisons of angular velocities recorded by each method using statistical parametric mapping. Red dashed lines represent critical thresholds for the t statistic for a given alpha level (0.025). Grey areas indicate regions that exceed the critical threshold. T statistics above zero indicate that the first method listed produces larger angular dorsiflexion velocities on average.

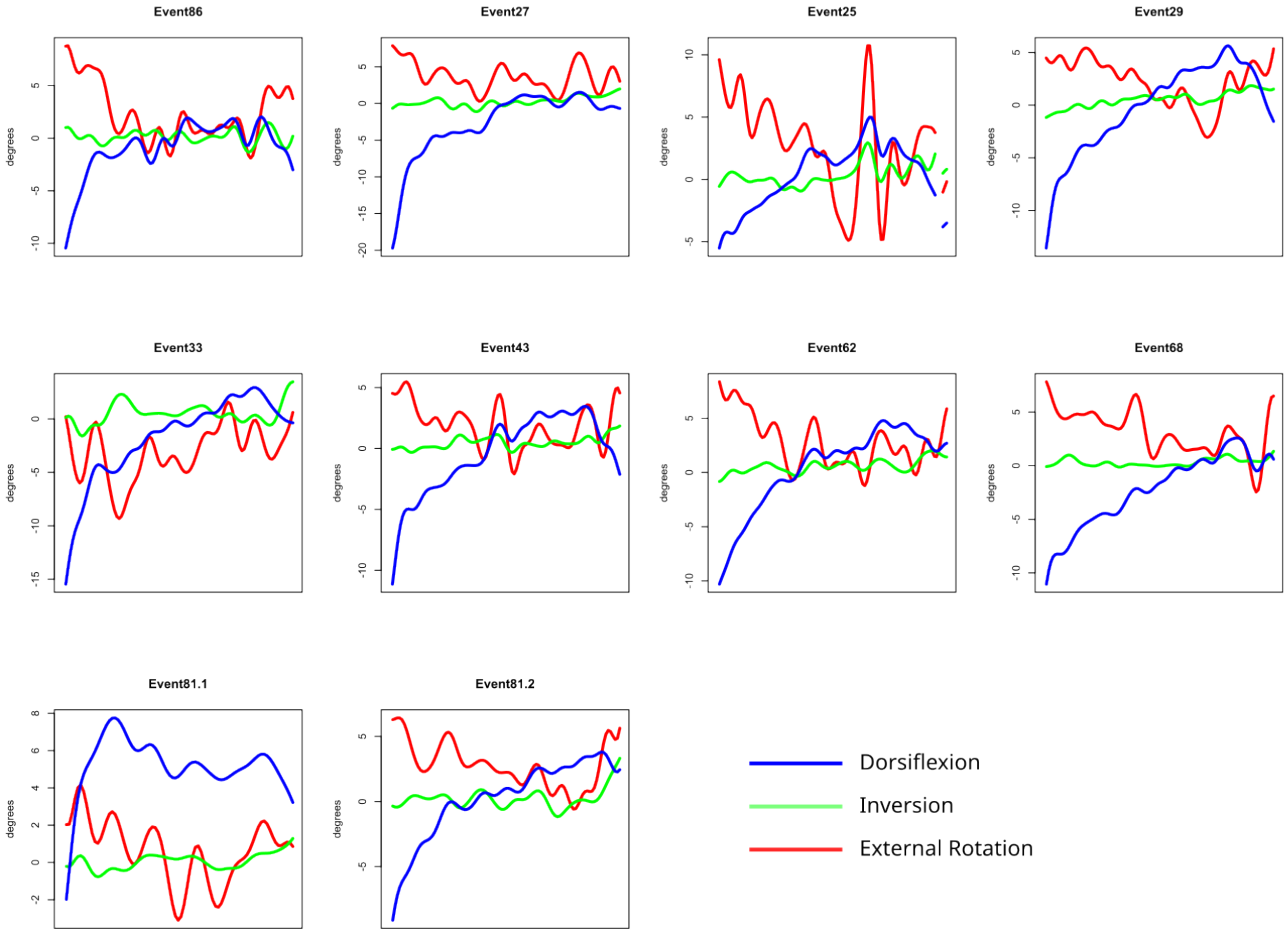

Fig. S8. Rotation across stance phase along all three axes. Blue indicates dorsiflexion, green indicates inversion, and red indicates external rotation. X-axis represents 0-100% of stance phase.

Journal of Experimental Biology • Supplementary information

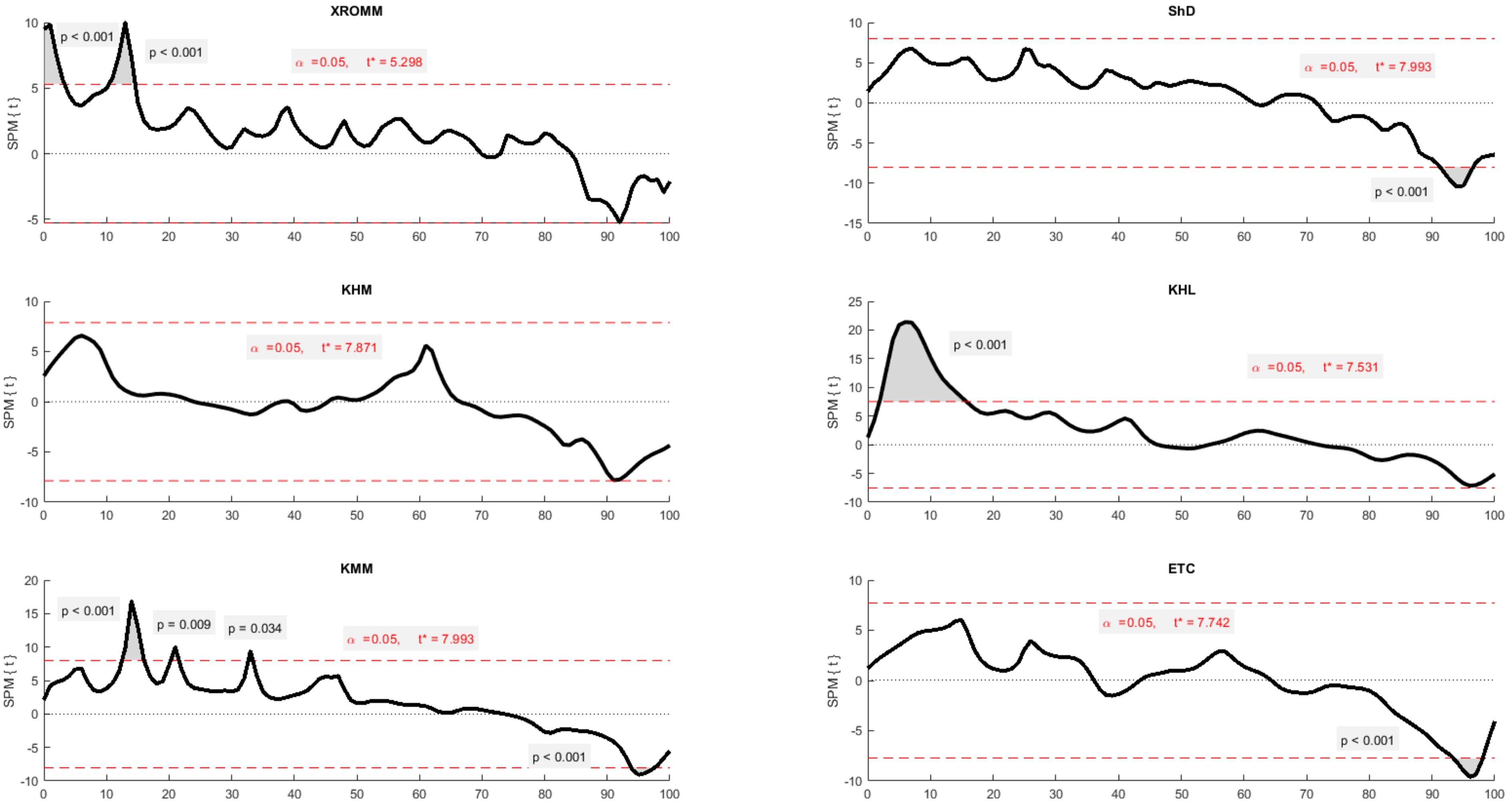

Fig. S9. One-way t tests using statistical parametric mapping to compare angular velocity to zero. Red dashed lines represent critical thresholds for the t statistic for a given alpha level (0.05). Grey areas indicate regions that exceed the critical threshold. T statistics above zero indicate angular velocity toward greater dorsiflexion, t statistics below zero indicate angular velocity toward greater plantarflexion.

Journal of Experimental Biology • Supplementary information

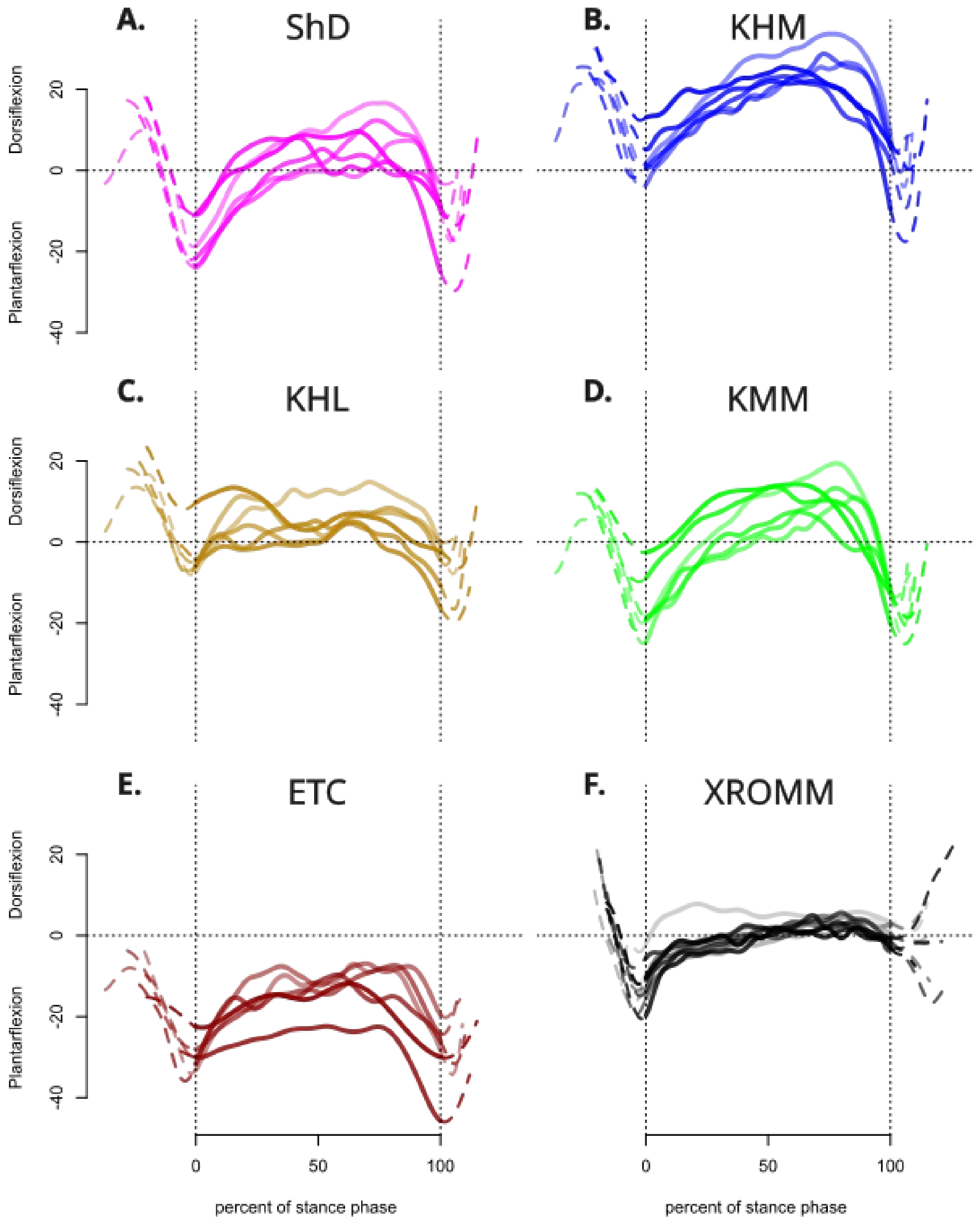

Fig. S10. All individual trials. Stance phase has been scaled but swing phase has not.

Journal of Experimental Biology • Supplementary information

Supplemental Table S1. Gait sequence and foot position relative to hand position across trials

Event	Gait Sequence	Foot Position Measured	Foot Position Other
Light 1.2	Diagonal	Lateral	Medial
Light 1.19	Diagonal	Medial	Medial
Light 1.22	Diagonal	Medial	Medial
Light 1.25	Diagonal	Medial	Medial
Light 1.28	Diagonal	Lateral	Medial
Light 1.29	Lateral	N/A	N/A
XROMM 2.25	Diagonal	Medial	-
XROMM 2.27	Diagonal	Medial	Medial
XROMM 2.29	Diagonal	Medial	Medial
XROMM 2.33	Diagonal	Medial	Lateral
XROMM 2.43	Diagonal	-	Medial
XROMM 2.62	Diagonal	-	-
XROMM 2.68	Diagonal	-	-
XROMM 2.81.1	Diagonal	Medial	-
XROMM 2.81.2	Diagonal	-	-
XROMM 2.86	Diagonal	Medial	Lateral

- Foot position could not be identified for all XROMM steps

Supplemental Table S2. Cutoff frequencies (Hz) used to filter each method and trial.

	Light 1.2	Light 1.19	Light 1.22	Light 1.25	Light 1.28	Light 1.29
ShD	9.3	10.5	12.1	9.3	7.6	11.3
KHM	14.2	18.7	13.8	18.7	15.1	14.4
KHL	8.3	8.9	14.0	9.1	7.8	14.0
KMM	8.3	8.1	14.1	12.5	14.4	10.5
ETC	5.6	5.7	10.4	7.4	9.3	12.0

Supplemental Table S3. Error across different methods of measuring angles

	Mean	SD	Min	Max
ShD	3.079	2.276	0.002	12.725
KHM	1.085	0.897	0.000	5.390
KHL	2.716	2.026	0.001	13.974
KMM	2.033	1.582	0.000	9.950
ETC	3.558	2.801	0.001	16.914
Comparisons (*p* values comparing grand mean and grand maximum error)				
ShD > KHM	**0.002**			**0.002**
ShD > KHL	0.394			0.240
ShD > KMM	**0.002**			0.041
ShD < ETC	0.180			0.485
KHM < KHL	**0.002**			**0.002**
KHM < KMM	**0.002**			**0.002**
KHM < ETC	**0.002**			**0.002**
KHL > KMM	0.026			0.180
KHL < ETC	0.041			0.132
KMM < ETC	**0.002**			**0.015**

Bold indicates p-value below 0.025.

Supplemental Table S4. p-values for comparisons across different methods of measuring dorsiflexion.

Comparison	Max dors. (unstandardized)	Max plant. (unstandardized)	Start stance (unstandardized)	Midstance (unstandardized)	End stance (unstandardized)	Max dors. (standardized)	Max plant. (standardized)	Midstance (standardized)	End stance (standardized)
ShD – XROMM	**0.0017**	**0.0075**	**0.0420**	0.0727	**0.0005**	**0.0002**	0.1806	**0.0002**	0.3676
KHM – XROMM	**0.0002**	**0.0030**	**0.0005**	**0.0002**	0.0727	**0.0002**	0.2198	**0.0005**	**0.0002**
KHL – XROMM	**0.0047**	0.3132	0.0160	0.1806	0.0559	0.4278	0.1179	**0.0225**	**0.0002**
KMM – XROMM	**0.0005**	**0.0075**	0.3132	**0.0160**	**0.0002**	**0.0002**	0.1471	**0.0002**	**0.0002**
ETC – XROMM	**0.0002**	**0.0002**	**0.0002**	**0.0002**	**0.0002**	**0.0420**	0.2198	0.0559	**0.0030**
ShD – KHM	**0.0022**	**0.0022**	**0.0022**	**0.0022**	**0.0152**	0.1320	> 0.999	0.2403	**0.0411**
ShD – KHL	0.9372	**0.0043**	**0.0022**	0.8182	0.3095	**0.0022**	0.8182	**0.0022**	**0.0260**
ShD – KMM	0.1797	0.4848	0.5887	0.3095	0.0649	0.6991	> 0.999	0.8182	0.0649
ShD – ETC	**0.0022**	**0.0022**	**0.0087**	**0.0022**	**0.0087**	**0.0022**	0.8182	**0.0022**	0.1320
KHM – KHL	**0.0022**	**0.0411**	**0.0411**	**0.0022**	**0.0260**	**0.0022**	0.5887	**0.0022**	0.1797
KHM – KMM	**0.0022**	**0.0022**	**0.0043**	**0.0022**	**0.0022**	0.0931	0.5887	0.3095	0.9372
KHM – ETC	**0.0022**	**0.0022**	**0.0022**	**0.0022**	**0.0022**	0.0931	0.8182	0.0649	0.9372
KHL – KMM	0.3095	**0.0043**	**0.0411**	0.2403	**0.0411**	**0.0022**	> 0.999	**0.0022**	0.0649
KHL – ETC	**0.0022**	**0.0022**	**0.0022**	**0.0022**	**0.0022**	0.0931	0.4848	**0.0152**	0.3095
KMM – ETC	**0.0022**	**0.0022**	**0.0043**	**0.0022**	**0.0043**	**0.0022**	0.4848	**0.0152**	0.9372
Kruskal-Wallis	$\chi^2 = 33.4$<br>*p* < **0.0001**	$\chi^2 = 31.6$<br>*p* < **0.0001**	$\chi^2 = 29.8$<br>*p* < **0.0001**	$\chi^2 = 29.2$<br>*p* < **0.0001**	$\chi^2 = 30.6$<br>*p* < **0.0001**	$\chi^2 = 30.4$<br>*p* < **0.0001**	$\chi^2 = 4.9$<br>$p = 0.427$	$\chi^2 = 30.5$<br>*p* < **0.0001**	$\chi^2 = 23.3$<br>*p* = **0.0003**

Dors = dorsiflexion; Plant = plantarflexion; ShD = Shank-Dorsum method; KHM = Knee-Heel-Metatarsal head method; KHL = Knee-Heel-Lateral margin method; KMM = Knee-Malleolus-Metatarsal head method; ETC = External TaloCrural method.

Journal of Experimental Biology • Supplementary information

Supplemental Table S5. Maximum angles and ranges of motion for each individual trial.

		XROMM Event 25	XROMM Event 27	XROMM Event 29	XROMM Event 33	XROMM Event 43	XROMM Event 62	XROMM Event 68	XROMM Event 81.1	XROMM Event 81.2	XROMM Event 86
XROMM	Maximum Dorsiflexion (stance)	5.0	1.5	5.6	2.9	3.5	4.8	2.6	7.8	3.8	2.0
	Maximum Plantarflexion (stance)	-5.5	-19.7	-13.6	-15.5	-11.1	-10.3	-11.1	-2.0	-9.1	-10.4
	Range of Motion (stance)	10.5	21.2	19.2	18.4	14.6	15.1	13.6	9.7	12.9	12.5
	Maximum Dorsiflexion (all measured)	18.7	23.3	10.5	4.1	3.5	21.0	10.9	8.0	9.9	8.1
	Maximum Plantarflexion (all measured)	-7.9	-20.5	-16.6	-19.5	-13.5	-11.9	-17.1	-12.1	-12.4	-12.1
	Range of Motion (all measured)	26.6	43.9	27.2	23.6	17.0	32.8	28.1	20.1	22.3	20.2
	Start of Stance	-5.5	-19.7	-13.6	-15.5	-11.1	-10.3	-11.1	-2.0	-9.1	-10.4
	Midstance	1.5	0.0	1.7	-0.3	1.3	1.8	-1.6	4.7	1.0	0.5
	End of Stance	-3.5	-0.7	-1.5	-0.4	-2.1	2.7	0.6	3.2	2.4	-3.0
		Light Event 02	Light Event 19	Light Event 22	Light Event 25	Light Event 28	Light Event 29				
ShD	Maximum Dorsiflexion (stance)	8.1	9.6	5.3	12.3	7.4	16.6				
	Maximum Plantarflexion (stance)	-24.9	-10.7	-21.5	-23.3	-24.0	-18.6				
	Range of Motion (stance)	33.0	20.4	26.8	35.7	31.4	35.3				
	Maximum Dorsiflexion (all measured)	8.1	17.9	13.4	17.3	9.8	18.0				
	Maximum Plantarflexion (all measured)	-29.7	-13.6	-22.0	-23.3	-24.0	-18.9				
	Range of Motion (all measured)	37.8	31.5	35.5	40.7	33.8	36.9				
	Start of Stance	-11.0	-10.7	-21.5	-23.3	-24.0	-18.6				
	Midstance	2.5	8.3	5.0	1.3	-0.1	9.5				
	End of Stance	-24.9	-8.9	-8.5	-9.8	-3.1	-9.9				
KHM	Maximum Dorsiflexion (stance)	22.0	25.5	23.2	28.8	26.6	33.7				
	Maximum Plantarflexion (stance)	-9.5	6.6	0.5	0.8	-3.5	1.8				
	Range of Motion (stance)	31.5	18.9	22.7	28.0	30.1	31.9				
	Maximum Dorsiflexion (all measured)	22.0	30.1	23.9	28.8	26.6	33.7				
	Maximum Plantarflexion (all measured)	-17.6	0.2	-9.5	-0.4	-4.4	-4.0				
	Range of Motion (all measured)	39.6	30.0	33.5	29.3	31.0	37.7				
	Start of Stance	5.1	13.0	0.5	1.3	-3.5	1.8				
	Midstance	21.9	24.0	22.7	19.8	18.5	28.1				
	End of Stance	-9.5	6.6	2.2	6.0	5.8	9.1				
KHL	Maximum Dorsiflexion (stance)	4.9	13.5	5.2	8.4	11.4	14.8				
	Maximum Plantarflexion (stance)	-16.2	-2.3	-10.7	-4.6	-5.7	-7.1				
	Range of Motion (stance)	21.1	15.7	15.9	13.0	17.1	21.9				
	Maximum Dorsiflexion (all measured)	4.9	23.4	19.6	18.0	13.5	16.7				
	Maximum Plantarflexion (all measured)	-19.6	-6.4	-16.5	-5.7	-7.1	-8.1				
	Range of Motion (all measured)	24.5	29.8	36.2	23.7	20.6	24.8				
	Start of Stance	-4.2	9.8	-5.7	-4.6	-5.7	-7.1				
	Midstance	-0.2	3.5	5.1	0.3	2.1	12.2				
	End of Stance	-16.2	-2.3	-10.7	-4.4	0.0	-0.7				
KMM	Maximum Dorsiflexion (stance)	13.7	14.2	7.5	13.3	11.1	19.4				
	Maximum Plantarflexion (stance)	-20.1	-12.6	-19.0	-18.5	-24.6	-19.7				
	Range of Motion (stance)	33.8	26.8	26.6	31.8	35.7	39.1				
	Maximum Dorsiflexion (all measured)	13.7	14.2	11.1	13.3	11.1	19.4				
	Maximum Plantarflexion (all measured)	-25.1	-18.7	-21.7	-18.5	-25.0	-22.6				
	Range of Motion (all measured)	38.9	33.0	32.9	31.8	36.1	42.0				
	Start of Stance	-8.9	-2.5	-19.0	-18.5	-24.6	-19.7				
	Midstance	13.4	13.5	4.7	3.2	1.0	11.7				
	End of Stance	-20.1	-12.6	-11.4	-12.7	-12.3	-12.8				
ETC	Maximum Dorsiflexion (stance)	-22.4	-11.8	-9.7	-7.4	-6.9	-7.0				
	Maximum Plantarflexion (stance)	-45.7	-29.9	-31.7	-28.0	-29.8	-33.1				
	Range of Motion (stance)	23.3	18.1	22.0	20.6	22.9	26.1				
	Maximum Dorsiflexion (all measured)	-22.4	-11.8	-9.7	-3.8	-6.9	-4.0				
	Maximum Plantarflexion (all measured)	-46.0	-30.2	-35.9	-28.0	-30.0	-34.3				
	Range of Motion (all measured)	23.6	18.3	26.2	24.3	23.1	30.3				
	Start of Stance	-30.0	-22.5	-31.7	-28.0	-29.8	-33.1				
	Midstance	-22.6	-14.8	-11.4	-14.0	-12.3	-11.6				
	End of Stance	-45.7	-29.9	-26.0	-24.2	-19.5	-28.2				

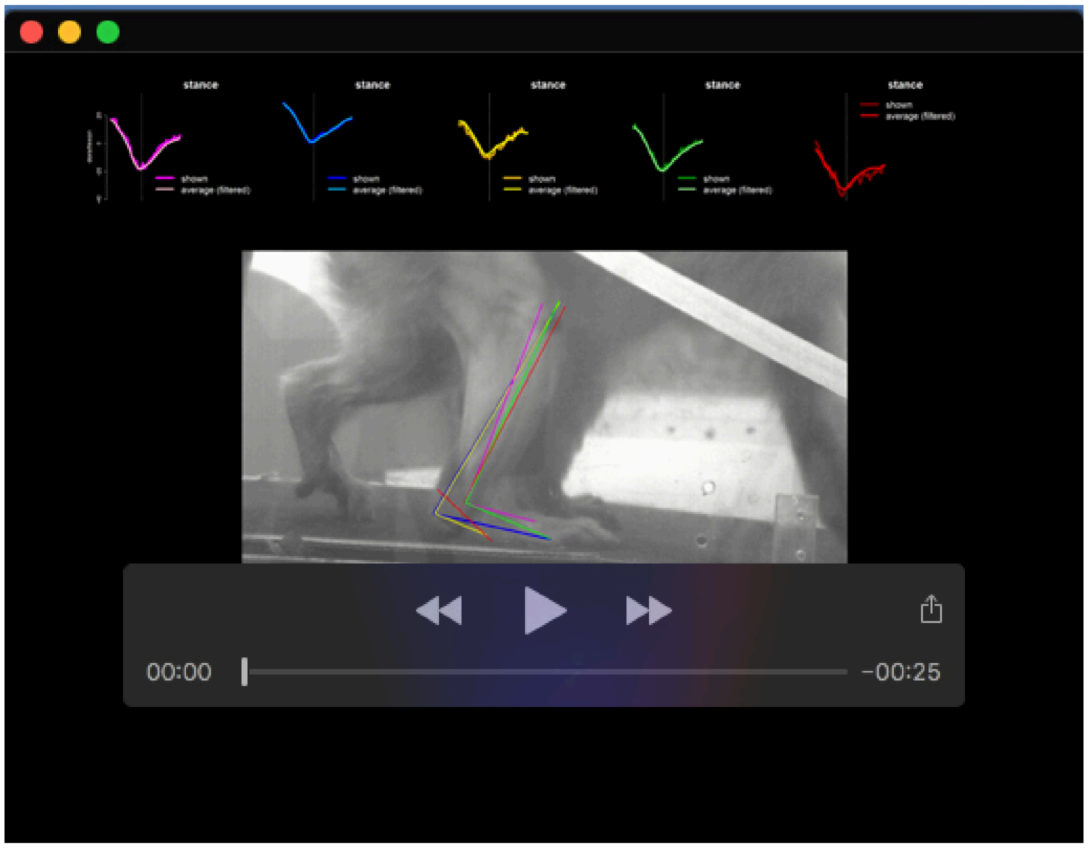

Movie 1. Video of light event 1.29 showing angles as they are measured in this study throughout a full step. Plots across the top show the angles. From left to right: ShD (magenta), KHM (blue), KHL (yellow), KMM (light green), ETC (red). Darker lines on the plots indicate the angle as illustrated in the video. Lighter lines on the plots indicate the angles used in analyses after averaging two measurements and filtering.

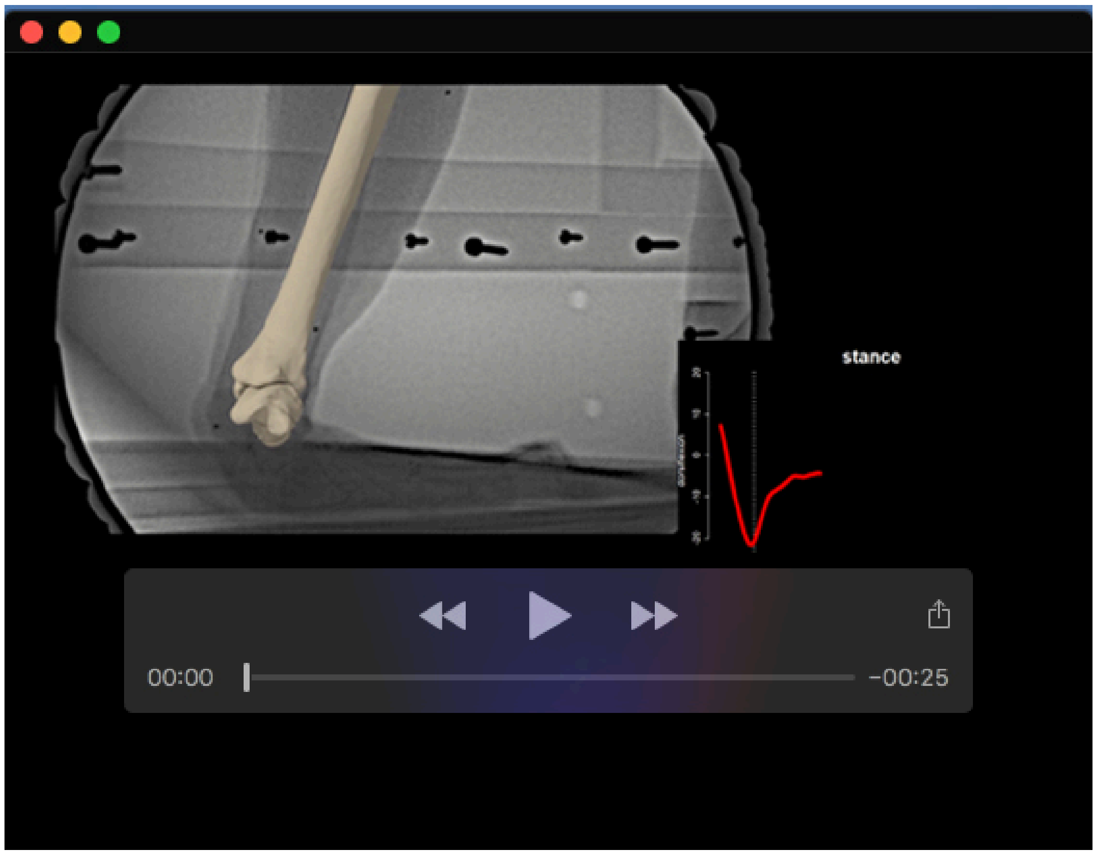

Movie 2. X-ray videos of XROMM event 2.27 showing the view through both cameras. Plot on the bottom right shows the angle of dorsiflexion at the talocrural joint.

Journal of Experimental Biology • Supplementary information
